# Bronchoconstriction: a potential missing link in airway remodelling

**DOI:** 10.1098/rsob.200254

**Published:** 2020-12-02

**Authors:** Michael J. O'Sullivan, Thien-Khoi N. Phung, Jin-Ah Park

**Affiliations:** Department of Environmental Health, Harvard T.H. Chan School of Public Health, 665 Huntington Ave, Boston, MA, USA

**Keywords:** asthma, bronchoconstriction, mechanical stress, airway remodelling

## Abstract

In asthma, progressive structural changes of the airway wall are collectively termed airway remodelling. Despite its deleterious effect on lung function, airway remodelling is incompletely understood. As one of the important causes leading to airway remodelling, here we discuss the significance of mechanical forces that are produced in the narrowed airway during asthma exacerbation, as a driving force of airway remodelling. We cover *in vitro*, *ex vivo* and *in vivo* work in this field, and discuss up-to-date literature supporting the idea that bronchoconstriction may be the missing link in a comprehensive understanding of airway remodelling in asthma.

## 1. Introduction

Globally, over 350 million people suffer from asthma [1–3]. Current treatments, which mainly target inflammatory cells, are less effective in some patients [4,5]. The limitations of the current therapies are due in part to the heterogeneity of the disease, which includes various endotypes [6–10]. While the root of asthma endotypes remains unclear, one of the major contributors appears to be airway epithelial cells [11,12]. A growing body of literature indicates that airway epithelial cells cause pathophysiological processes when functionally altered by mechanical forces generated in the narrowed airway during bronchoconstriction [13–17]. This review highlights the evidence supporting the idea that the mechanical force imposed on the airway epithelium is an important contributor to the disease progression in asthma.

## 2. Bronchoconstriction: cause or effect?

In patients with asthma, a variety of external insults, including environmental pollutants [18], bacteria [19] and viruses [20], frequently cause exacerbations, which present clinically as increased inflammation and bronchoconstriction [21–23]. Thus, bronchoconstriction has been regarded as a consequence or manifestation of airway remodelling attributable to inflammation during the progress of disease. During bronchoconstriction, the airway becomes narrowed, the airway lumen is squeezed and consequently airway epithelial cells lining the lumen are mechanically compressed [24,25]. Data now indicate that mechanically compressed airway epithelial cells resulting from bronchoconstriction might reawake or propagate the pathologic process and lead to airway remodelling and eventually to repeated exacerbations [13,16,26–28]. These observations suggest, in turn, that bronchoconstriction may not simply be a manifestation but could also be a cause of airway remodelling. If bronchoconstriction is proven to be a driver of airway remodelling, preventing asthma exacerbations could be a reasonable approach to mitigate worsening of the disease. If reducing asthma exacerbations mitigates disease progression, this will further highlight the importance of prevention of exacerbation in the clinic.

## 3. Airway epithelial cells during bronchoconstriction

Until the late 1990s, the impact of mechanical forces on airway disease remained largely unknown. Felix and colleagues performed pioneering experiments demonstrating that airway epithelial cells respond to mechanical forces [29,30]. Their data revealed that stretch causes the airway epithelial cells to release inositol trisphosphate [29] and that mechanical stimulation with a micropipette leads to calcium mobilization [30]. However, in these studies using of tissues understanding epithelial responses to mechanical forces at a cellular level was limited.

In an effort to overcome this limitation, a new culture technique was developed, in which primary hamster tracheal epithelial cells were grown on a porous surface in a biphasic chamber [31]. Following confluency of the cells, establishing an air-liquid interface (ALI) allows airway basal stem cells to differentiate to a polarized, pseudostratified epithelium that is similar to the cellular composition of the intact *in vivo* airway. The culture contains differentiated cells, including goblet and ciliated cells [32–34]. This ALI culture of airway epithelial cells substantially improved the knowledge of airway epithelial cell biology, including cellular responses to mechanical forces [13–16,27,35–40]. Using this system, one of the earliest studies investigating the impact of mechanical force on the cellular responses identified that mechanical forces induce the release of extracellular nucleotides, including ATP and UTP, which depends on increased intracellular calcium concentration [36]. The generation of calcium waves within airway epithelial cells might act as defence mechanisms on airway surfaces [41]. As the evidence accumulated, it became better appreciated that epithelial cells actively respond to mechanical cues, although at the time, the biological consequence of these responses was unknown.

### 3.1. Activation of biological signals by mechanical compression *in vitro*

The first evidence of the pathophysiological impact caused by mechanical forces on airway epithelial cells emerged by mimicking mechanical forces exerted to the airway epithelium during asthma exacerbations. During this bronchoconstriction, the airway becomes narrowed and the tissue on the luminal side of the airway becomes buckled. In this buckled tissue, the airway epithelial cells become squeezed [24], experiencing a compressive, mechanical stress. This compressive stress during maximal airway constriction was computed to be on the order of magnitude of 30 cm H_2_O [24]. To mimic the effect of the squeezed epithelium, this computed mechanical force was applied to rat tracheal epithelial (RTE) cells in ALI culture. Using a unique mechanical compression system, the cells were exposed to an apico-basal pressure gradient. Ressler and colleagues demonstrated that mechanical compression with 20 cm H_2_O pressure induces the mRNA expression of early growth response-1 (Egr-1), endothelin (ET-1) and TGF-β1 [42] in RTE cells. Following these initial studies, well-differentiated human bronchial epithelial (HBE) cells were adopted as the gold standard model for *in vitro* studies of airway epithelium. When HBE cells are exposed to pressure at a magnitude of 30 cm H_2_O, spatial deformation of compressed epithelial cells occurs and the lateral intercellular space (LIS) between adjacent cells is reduced [35]. Importantly, as the total volume of LIS is reduced, local concentrations of receptor ligands released by the adjacent cells are increased [35]. The increased EGFR ligands, including HB-EGF, cause the activation of ERK signalling [28]. In asthma, EGFR signalling has been linked to a variety of pathophysiologic processes, including goblet cell hyperplasia (GCH) [43–45] and secretion of inflammatory mediators [46]. Interestingly, EGFR-dependent GCH was observed in the HBE cells exposed to mechanical compression [13] (table 1). This evidence suggests that bronchoconstriction alone is sufficient to increase the number of goblet cells, which are responsible for mucus hypersecretion causing airway obstruction during asthma exacerbations. One of the secreted mediators induced by mechanical compression is YKL-40, a protein that is expressed in the airway epithelium [15]. Secretion of YKL-40 that is induced by mechanical compression depends on EGFR signalling [15]. In patients with asthma, increased serum levels of YKL-40 are correlated with disease severity, airway remodelling and decreased pulmonary function [49,50]. YKL-40 has been shown to induce the production of pro-inflammatory cytokines, including IL-8 from cultured HBE cells and BEAS-2B cells [51] and to induce proliferation and migration of BEAS-2B cells [52]. These studies investigating the role of YKL-40 in bronchial epithelial cells used submerged cells. To increase the physiological relevance of these earlier studies, examining the effect of YKL-40 on well-differentiated HBE cells will be necessary.

**Table 1.** Studies demonstrating the impact of bronchoconstriction on airway remodelling.

	recapitulated features of airway remodelling by experiments		
features of airway remodelling	*in vitro* compressive system	PCLS Contraction	bronchoconstriction *in vivo*
inflammation	possible	not determined	not detected [26] increased macrophages [47]
subepithelial collagen deposition	collagen type III [27]	probable (given TGF-β release [48])	collagen type III [26]
goblet cell hyperplasia	MUC5AC [13]	not determined	PAS staining [26]
airway smooth muscle-cell proliferation and contraction	increased proliferation, increased contraction [16]	increased contractile phenotype marker protein [48]	not observed [47]

While the activation of the EGFR by mechanical compression is the most well studied, the activation of other intracellular signalling pathways by mechanical compression is also reported [15,38]. In airway epithelial cells, protein kinase C (PKC) is known to regulate a variety of remodelling events, including mucin hypersecretion [53] and secretion of NF-κB-dependent inflammatory cytokines [54]. In HBE cells, mechanical compression induces the activation of PKC as detected by the phosphorylation of MARCKS, a PKC substrate [14,15]. Furthermore, blocking PKC activation attenuates the release of tissue factor and maspin, both of which are induced by compression [14,38]. Both tissue factor and maspin are increased in the patients with asthma and after allergen challenges in mice and humans [14,38,39,55]. Together with the evidence demonstrating the activation of EGFR and PKC by mechanical compression, it is clear that multiple signalling cascades become activated upon mechanical compression of the airway epithelium. Further studies targeting other signalling pathways will lead to a better understanding of the changes that occur in the asthmatic airway wall. In particular, intracellular signalling pathways regulating the secretion of pathologic mediators into distinct extracellular spaces of the airway could represent potential targets toward the treatment of asthma. In the following section, we will discuss the role of the secreted mediators from compressed HBE cells in airway remodelling.

### 3.2. Airway remodelling recapitulated by mechanical compression *in vitro*

Mechanical compression of airway epithelial cells leads to remodelling of the epithelium itself, but also leads to changes to the extracellular matrix (ECM) of neighbouring cells. Swartz and colleagues demonstrated the potential for the compressed epithelium to remodel the airway ECM [27]. After incubation of airway fibroblasts with conditioned medium transferred from compressed HBE cells, the fibroblasts release increased collagen type III [27] (table 1). In this study, there was no direct contact between epithelial cells and fibroblasts. Thus, epithelial-derived soluble factors might lead to a synthetic fibroblast phenotype, which is characterized by increased connective tissue synthesis. In addition, pro-fibrotic mediators, endothelins and TGF-β2, are also increasingly secreted by compression of HBE cells [16,56], suggesting mechanical compression might activate fibrotic signalling in the airway epithelial cells.

Mechanical compression of the airway epithelium initially results from the airway smooth muscle (ASM) contraction. Interestingly, data revealed that smooth muscle contraction can be augmented by compressed epithelial cells. Lan and colleagues demonstrated that conditioned medium from compressed HBE cells leads to an increased ASM contraction that is measured by traction forces [16] (table 1). ASM contraction that is induced by the conditioned medium from compressed epithelial cells depends on endothelin receptor signalling. The compressed epithelial-derived conditioned medium also induces proliferation of ASM cells. Both ASM hypercontraction and hyperproliferation represent mechanisms by which ASM contributes to the pathology found in the asthmatic airway. Therefore, it is critical to understand what regulates these processes to uncover new ways to treat this disease.

A recent study using RNA sequencing analysis reveals that transcriptomic profiles of non-asthmatic cells exposed to compression recapitulate the genetic signature of unperturbed asthmatic cells [17]. These data provide new insights into the potential molecular mechanisms by which non-asthmatic cells become genetically similar to the cells found in the asthmatic airway. In particular, the data highlight that mechanical compression itself is sufficient to induce inflammatory, repair and fibrotic signalling pathways, all of which are associated with the pathogenesis of asthma. Interestingly, some of the genes and pathways highly enriched in this new analysis overlap with our previous findings [57]. Moreover, gene ontology analysis indicates that compression-induced genes are associated with focal adhesions and cytoskeleton, both of which are responsible for the regulation of intercellular forces. This new analysis provides potential mechanisms by which mechanical compression induces epithelial cell unjamming as described in the following section. These genomics approaches assure that compressed epithelial cells undergo pathophysiologic and physical changes, both of which play critical roles in the pathophysiology of asthma.

### 3.3. Airway epithelial unjamming by mechanical compression

**Figure 1.**
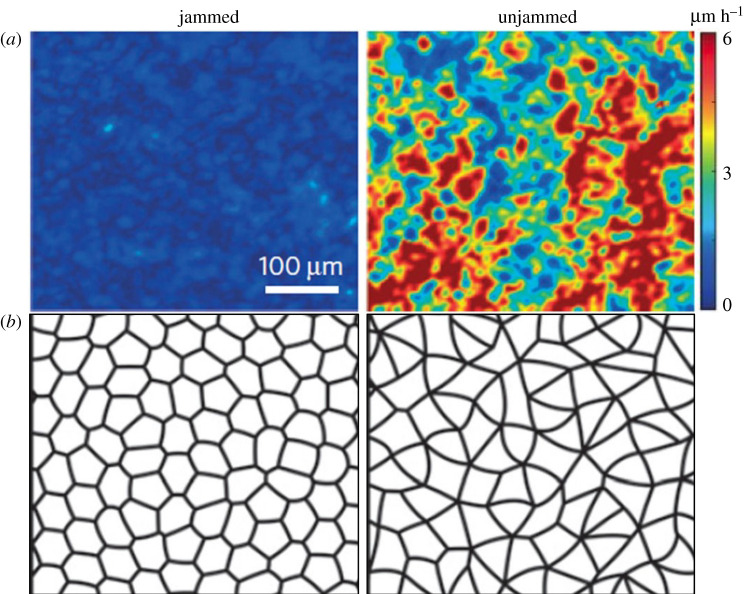
Increased cellular velocity and cell shape elongation are characteristics of the unjammed layer. (*a*) Representative speed maps show the differences in cellular velocities between jammed and unjammed layers. The cellular velocity is increased in the unjammed layer, which is induced by mechanical compression. (*b*) The theory developed by the vertex model predicted cell shape changes during the phase transition between the jammed and unjammed layers. Predicted cell shape is cobblestone-like in the jammed layer, while it is elongated in the unjammed layer. Figure from [40] with the permission from the publisher.

In addition to extensive efforts studying biochemical and transcriptional regulation of dysregulated asthmatic epithelia, physical changes to cells have also been explored. The physical behaviours of cells were studied using primary HBE cells grown in ALI, where they differentiate and recapitulate a pseudostratified epithelium as in the airway *in vivo* [32–34]. When HBE cells are mature and well-differentiated, as those found in intact tissues, the cellular collective remains non-migratory like a solid, which is termed a jammed phase. When the cells in this jammed layer are exposed to mechanical compression that mimics mechanical forces as described above, the cellular collective becomes migratory like a fluid, which is termed an unjammed phase. During this transition of the cellular collective from the jammed to the unjammed phase, which is termed an unjamming transition (UJT), the cellular motility is increased, and the cell shapes become elongated (figure 1) [24,35,40,57]. While discovering the UJT marked by cellular motility in the experimental system (figure 1*a*), a novel metric-based on cell shape was proposed to predict UJT based on a theoretical computational model of epithelial cells (figure 1*b*). This cell shape elongation predicted by the theoretical model has been further validated in the experimental data [40,58–60].

**Figure 2.**
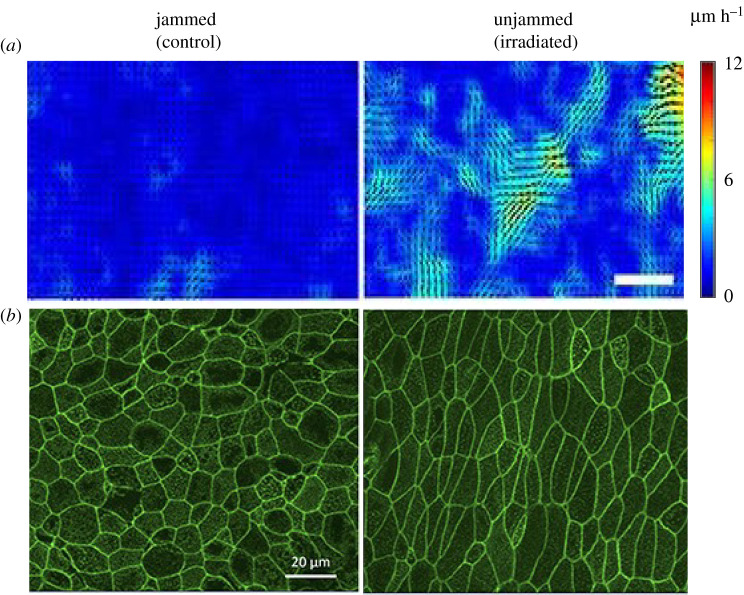
Unjamming transition is induced by ionizing radiation. (*a*) Representative speed maps show the differences in cellular velocities between jammed and unjammed layers. The cellular velocity is increased in the unjammed layer, which is induced by ionizing radiation. Scale bar, 100 μm. (*b*) Phalloidin staining from the corresponding cells above show the differences in cell shape between jammed and unjammed layers. HBE cells maintain a cobblestone-like shape in the jammed layer, whereas become elongated shape in the unjammed layer. Figure from [59] with the permission from the publisher.

In well-differentiated HBE cells, the UJT is also induced after exposure to repeated ionizing radiation [40]. In control wells, the layer is jammed, and the cells maintain a cobblestone-like shape, whereas in irradiated wells the layer becomes unjammed, and the cells become elongated in shape (figure 2*b*).

Interestingly, this unjammed phase is also observed when cells are immature and less differentiated in ALI culture. In conditions where HBE cells are mature and well-differentiated, the cellular layer becomes jammed. This jamming transition is delayed in the cells cultured from asthmatic donors, suggesting that the unjammed phase might represent a dysregulated phenotype of the asthmatic or injured epithelium. Although the mechanisms that cause the jamming and UJT of the epithelium remain unknown, one potential mechanism underlying the UJT could be the activation of the TGF-β receptor. In HBE cells, blocking of the TGF-β receptor activity partially attenuated the irradiation-induced UJT [59]. One could hypothesize that the increased cellular motility during the UJT is due to the epithelial-to-mesenchymal transition (EMT) through the activation of the TGF-β receptor. However, signs of EMT were not observed during the UJT in HBE cells [59,61]. Furthermore, the biochemical and biophysical characteristics of the cellular migration between the EMT and UJT are distinct [61].

Since the discovery of the UJT in the HBE cells, it has been increasingly recognized and observed in multiple *in vitro* and *in vivo* systems in the context of embryonic development, wound healing and cancer metastasis [58,62–64]. During development, the UJT might be a necessary process for collective cellular migration, but under pathologic conditions, including asthma and cancer, it may represent dysregulated cellular phenotypes linked to dysregulated cellular functions. The role of the UJT in disease remains to be understood.

## 4. The impact of mechanical forces assessed in tissues

To extend *in vitro* findings using airway epithelial cells to systems in which multiple types of lung cells are contained, *ex vivo* approaches have emerged. In one of the earlier approaches, the whole lung isolated from rats was used to investigate the effect of cyclical increases in airway and vascular pressures on the transcriptional changes of ECM proteins such as procollagen, laminin and fibronectin [65]. mRNA expression of the ECM proteins is increased in whole isolated lungs exposed to high positive end-expiratory pressures for four hours, suggesting that excessive forces applied to the tissues modulate extracellular environment of the lung by transcriptional regulation. In a similar *ex vivo* approach using whole isolated mouse lung, the activation of EGFR was detected in the airway epithelium in the constricted airway that was established by a tracheal perfusion with methacholine [35]. The activation of EGFR marked by p-EGFR is also detected in the mechanically compressed HBE cells [35]. Approaches using whole lung provide a broader view of pathophysiologic changes that occur by mechanical force in the lung. Despite the advantage of using whole lung, it has not been widely used because of technical limitations. A new approach was introduced using precision cut lung slices (PCLS), which possess many advantages including the capability of mechanistic studies in multiple regions of the lung isolated from multiple species, including rodents [66–71], non-human primates [72,73], and humans [74–78]. The constricted PCLS that is induced by methacholine exposure reproduce the dynamic and mechanical events of the human airways during constriction [79], supporting the notion that PCLS can be a relevant model to study the impact of mechanical forces on lung physiology. For example, PCLS can be used as a model for both airway constriction and lung stretch [69,70]. When PCLS were constricted by either TGF-β1 or methacholine, the expression of contractile proteins was increased, similar to those detected as in the remodelled airway in asthma [48] (table 1). Moreover, methacholine-induced expression of contractile proteins depends on the TGF-β receptor, suggesting a potential role of released TGF-β upon airway constriction [48]. When PCLS were stretched mechanically, the activation of the biochemical signalling cascades, including calcium mobilization, was observed [69]. The use of PCLS has been further advanced by the incorporation of traction force microscopy that can quantify the force responses to a variety of stimuli [80]. Although PCLS are a powerful and versatile tool to investigate the role of mechanical force in lung physiology, there are some limitations worth noting. For example, the use of PCLS takes place in submerged culture, which does not recapitulate the air-liquid environment in the lung. In addition, the slice of tissues excludes the interaction with circulating leukocytes and inflammatory cells available in other *in vitro* systems.

## 5. The impact of mechanical forces assessed *in vivo*

The *in vitro* findings described above were validated by Grainge and colleagues, indicating that mechanical compression leads to the major events of airway remodelling [26] (table 1). In their study performed in humans, repeated, experimentally induced bronchoconstriction led to GCH and thickening of the basement membrane. This study provided further evidence that bronchoconstriction itself is sufficient to induce airway remodelling, in the absence of infiltrated inflammatory cells to the airway. Except this study, most *in vivo* studies investigating the role of bronchoconstriction on airway remodelling have been conducted using rodents. In rats, excessive mechanical ventilation, deemed as a positive end-expiratory pressure of 1.5 cm H_2_O, caused an increase in proteoglycan ECM around the airways, [81] suggesting that mechanical strain might cause matrix remodelling in the airway. In guinea pigs, ASM remodelling is inhibited by the airway relaxation that is mediated by muscarinic receptor antagonist prior to challenge with ovalbumin (OVA) [82]. This study suggests that during the bronchoconstriction associated with OVA, epithelial-derived mediators induce ASM-cell proliferation, which can be attenuated by muscarinic antagonism. Similar to the study performed in humans, the impact of repeated bronchoconstriction was investigated in mice. Indeed, as in humans, repeated bronchoconstriction leads to goblet cell metaplasia in mice [47]. It is still unknown whether repeated bronchoconstriction leads to altered lung function in human, but repeated bronchoconstriction did not cause reduced lung function in mice [47]. Repeated exposure to methacholine following sensitizations and challenges with OVA leads to prolonged or augmented airway inflammation and remodelling [83], suggesting the role of bronchoconstriction in the potentiation of disease progress in asthma. While mice have been widely used for understanding asthma, we also need to recognize anatomical and physiological differences between humans and mice [84–86]. In a mouse model recapitulating asthma-like phenotypes, one of the key features of chronic asthma in humans, remodelling of ASM, is either not observed or observed only after high level challenge, suggesting that mechanisms underlying bronchoconstriction could possibly be distinct between mice and humans or the degree of bronchoconstriction in mice could possibly be lower than in humans [85]. For example, in a mouse model of asthma, AHR is not due to increased ASM shortening but can be due to airway closure caused by a thickened mucosa [87]. Furthermore, another study showed that porcine airways secreted more mucus when challenged for a second time with cholinergic stimulation, indicating the importance of repeated airway narrowing in leading to the production of mucus [88]. However, it should be noted that the second challenge in this study was administered in an environment of reduced chloride and bicarbonate transport to mimic cystic fibrosis [88]. Future work to unravel how the airway epithelium in constricted airways propagates airway inflammation and remodelling should carefully consider the physiological relevance of species and models to be used.

## 6. Remaining questions: crosstalk between mechanical force and exacerbation factors

The data clearly indicate that mechanically compressed airway epithelial cells play a causal role in airway remodelling and asthma. However, airway epithelial cells are also influenced by pre-existing inflammatory conditions or preceding stimuli provoking exacerbations. Among the factors provoking asthma exacerbations, including volatile chemicals, allergens, environmental pollutants, and bacterial and viral infections [89,90], the most frequent cause of asthma exacerbation is infection with respiratory viruses such as respiratory syncytial virus and rhinovirus (RV) [91,92]. Epidemiological data indicate the association between viral infections and severity of asthma symptoms [93]. RV infections cause 57% of asthma exacerbations in adults [94] and 85% of asthma exacerbations in children [92]. Furthermore, children who are exposed to both respiratory allergens and RV, are ten times more likely to develop wheezing illnesses than children who are not exposed to either insult [95]. Despite the fact that asthma exacerbations are presented by both inflammation and bronchoconstriction, most studies examine the role of inflammation, rather than bronchoconstriction. Thus, little is known about how airway epithelial cells integrate pathologic signals initiated by the viral infection and mechanical compression during asthma exacerbations induced by viral infection. A recent study showed that mechanical compression of HBE cells from asthmatic subjects causes the cells to produce a lower level of anti-viral interferon (IFN) in response to RV infection, compared to uncompressed cells [96,97], indicating that mechanical compression can modulate anti-viral immunity of the airway epithelial cells. This finding may provide an explanation to why asthmatic HBE cells fail to mount a protective anti-viral response to RV infections [98]. There may be an interaction of signalling events activated by compressive stress that impairs the ability of the cells to produce anti-viral cytokines, IFNs. For a better understanding of the crosstalk between previously discussed stimuli, such as RV infection and mechanical compressive stress, and the progression of the disease, further mechanistic studies are required. In addition, co-exposure of the airway to mechanical compression along with other features of the disease, such as airway inflammation, are likely to combine and lead to deleterious airway remodelling and these combination exposures should be considered in future research.

## 7. Conclusion

Asthma is a disease that has been recognized by remodelling of the airway wall since the 1900s [99–101]; however, our understanding of asthma pathogenesis is still lacking and slowly growing. Both chronic inflammation in the airway and reversible bronchoconstriction are cardinal features of the disease. In targeting these two critical symptoms, common treatment strategies include anti-inflammatory corticosteroids and beta-adrenergic receptor agonist *following* the onset of exacerbation. However, a growing body of literature now suggests that bronchoconstriction itself is sufficient to cause pathophysiological changes leading to airway remodelling. That is to say that bronchoconstriction itself turns on the pathologic signals leading to the progression of asthma. We therefore must create treatment strategies that are preemptive rather than reactive or responsive. To support this idea, here we discussed recent evidence indicating that bronchoconstriction facilitates the pathologic process through the activation of the airway epithelial cells. With the evidence from multiple approaches, including transcriptional and translational regulation, signalling pathways, RNA-sequencing analysis and collective cellular migration, it is evident that mechanically compressed airway epithelial cells play a causal role in the pathophysiology of asthma [13–17,35,38,39,61].
